# Yoga in Pediatric Gastroenterology

**DOI:** 10.1007/s11894-024-00941-9

**Published:** 2024-08-13

**Authors:** Francis Peropat, Mazen I. Abbas, Maria E. Perez, Elizabeth L. Yu, Alycia Leiby

**Affiliations:** 1 Atlantic Children’s Health-Goryeb Children’s Hospital, Morristown, NJ USA; 2 Kapi’olani Medical Center for Women and Children, Pediatric Gastroenterology, Honolulu, HI USA; 3 Division of Pediatric Gastroenterology, Hepatology, and Nutrition, Ann and Robert H. Lurie Children’s Hospital, Chicago, IL USA; 4 Department of Pediatrics, Northwestern University Feinberg School of Medicine, Chicago, IL USA; 5 Division of Gastroenterology, Hepatology and Nutrition, Rady Children’s Hospital, San Diego, CA USA; 6 Department of Pediatrics, University of California, San Diego, CA USA; 7 Atlantic Children’s Health-Goryeb Children’s Hospital, Pediatric Gastroenterology and Nutrition, Morristown, NJ USA; 8 Department of Pediatrics, Sidney Kimmel Medical College of Thomas Jefferson University, Philadelphia, PA USA

**Keywords:** Yoga, Integrative Medicine, Children, Inflammatory Bowel disease, Irritable Bowel Syndrome

## Abstract

**Purpose of Review:**

Pediatric use of yoga as an integrative medicine modality has increased in prevalence over the last several decades. In this article, we review the available evidence for yoga in pediatric gastrointestinal disorders.

**Recent Findings:**

Evidence supports that in many pediatric disorders of gut brain interaction (DGBI), including irritable bowel syndrome, functional abdominal pain and functional dyspepsia, yoga decreases pain intensity and frequency and increases school attendance. Yoga has been shown to improve health-related quality of life and improve stress management as an effective adjunct to standard medical therapy in pediatric inflammatory bowel disease (IBD). Further studies are needed regarding optimal frequency, duration of practice and evaluation of the impact on IBD disease activity measures.

**Summary:**

Yoga may benefit pediatric gastroenterology patients with DGBIs and IBD through improving quality of life and reducing pain. Future yoga studies could investigate biomarkers and continued research will help integrate this modality into routine pediatric gastroenterology care.

## Introduction

Pediatric integrative medicine, which combines complementary and alternative therapies (CAM) with conventional medicine, embodies a holistic approach to quality medical care and is gaining more acceptance by patients, parents and healthcare professionals. The field has been growing organically as pediatric chronic health conditions rates have been rising over the past three decades and are contributing to a larger burden of disability [1]. Childhood use of CAM in the United States was reported to be 11.6% according to the 2012 National Health Interview Survey (NHIS) [2]**.** However, more recent studies have shown a considerably higher rate of use of CAM among children with common modalities including dietary supplements, osteopathy, chiropractic, naturopathy, homeopathy, massage therapy, herbal medicine, Traditional Chinese medicine, Ayurveda, and mind–body medicine [3]. A meta-analysis of 20 worldwide studies reported 23.0% of pediatric patients used CAM short-term (≤ 12 month) and 77.7% in their lifetime for a variety of indications with respiratory conditions and gastrointestinal complaints being the most common [4]. Mental health issues in children such as depression and anxiety are another primary reason for CAM use and frequently co-exist with other chronic medical conditions [5]. Pediatric patients with digestive diseases are using several CAM methods with yoga emerging as an effective modality helpful in both functional gastrointestinal disorders such as irritable bowel syndrome (IBS) [6–10] as well as inflammatory bowel disease (IBD) [11–16] (Table 1).

**Table 1:** Studies of yoga in pediatric gastroenterology

Titles and Authors	Aims	Intervention	Results
Yoga as adjunct therapy for adolescents with IBD: A pilot clinical trial. (Arruda, JM, et. al Complement Ther Med 2018)	To determine whether utilizing yoga as adjunctive therapy to conventional medical care in adolescents with IBD is feasible, safe and acceptable	Prospective 8-week study combined in-person and video-based yoga intervention study period: 3 × of 60 min in-person yoga classes (weeks 1, 3, 8) and 3x/week 30-min online yoga classes *n* = 9 Age: 10–21-year-olds with diagnosis of IBD 2/9 patients completed all 3 on-line videos/week	Decrease in stress, increase in emotional self-awareness and ability to manage physical symptoms of IBD Limitations: Lack of power to see changes in PUCAI, calprotectin, or PROMIS-37 domains, difficulty completing yoga videos due to time limitations
A prospective, controlled multisite trial of yoga in pediatric IBD. (Leiby A, et al. JPGN 2023)	To evaluate if a structured yoga program improves HRQOL (health-related QOL) and self-efficacy in pediatric IBD patients	Prospective, multi-site, 12 week, in-person Iyengar yoga intervention at 2 clinical sites, classes offered once a week with encouragement to practice daily at home *n* = 78 Age: 10–17-year-olds with IBD 56/78, 72% completed 9 + classes	Significant improvements in HRQOL and general self-efficacy, even 3 months after conclusion of yoga classes. 85.2% of participants said yoga helped them to control stress Limitations: Non-randomized, baseline HRQOL relatively good. Potential volunteer bias
Iyengar yoga for adolescents and young adults with IBS (Evans S, et al. JPGN 2014)	To investigate the impact of Iyengar yoga on IBS symptoms	6 weeks, 2 × per week yoga classes *n* = 51 participants Age: 14–26 yrs Variables measured included IBS symptoms, health related quality of life, psychological distress, fatigue and sleep	Adolescents assigned to yoga had improved physical functioning Young adults in the yoga group endorsed improved IBS symptoms, global improvement, disability, psychological distress, sleep quality, and fatigue Limitations: Lacked an active control group, differential attrition between the groups
A randomized trial of yoga for adolescents with irritable bowel syndrome (Kuttner L, Pain Res Manage et al. 2006)	To evaluate yoga as a treatment for IBS, would reduce pain, GI symptoms, and functional disability	Randomized, controlled trial 4 weeks, daily home practice after one in person program *n* = 25 Age: 11–18 yrs Both groups completed questionnaires pre and post intervention	Participants in yoga group reported lower levels of functional disability, gastrointestinal symptoms, and anxiety Limitations: Sample size
A pilot study of yoga treatment in children with functional abdominal pain and irritable bowel syndrome (Brands M, et al. Complement Ther Med, 2011)	Investigate yoga exercises on frequency and intensity of pain with children with functional abdominal pain	10 yoga lessons and a pain diary *n* = 20 Age: 8–18 yrs Additionally, they were scored using Kidscreen quality of life questionnaire	Significant improvement in pain frequency and intensity and quality of life Limitations: Sample size
Yoga Therapy for Abdominal Pain-Related Functional Gastrointestinal Disorders in Children: A Randomized Controlled Trial (Korterink J, et al. JPGN 2017)	To evaluate effects of standard medical care (SMC) with yoga therapy compared to SMC on quality of life and abdominal pain	10 weeks of once weekly, 1.5 h. yoga therapy *n* = 69 Age: 8–18 yrs Scored on pain frequency and quality of life and followed for 12 months	Significant reduction in school absence and improved abdominal pain at 12 weeks. No differences were found for quality of life when comparing SMC and yoga therapy to SMC Limitations: Differential attrition between the groups

Yoga is an ancient practice that has its origin in the Indian traditional healthcare system of Ayruveda and is increasingly popular in Western nations. Yoga, meaning to “yoke” or “join” in Sanskrit, is a philosophy that seeks to “yoke” or unite the mind, body, and spirit. The practice has many components, including breathing exercises (pranayama), specific stretching movements that allow for body awareness and conditioning (asana), and meditation with presence (dhyana) [10]. Yoga practice has been shown to have mental health benefits in adults and children [5] and has shown that children have improved functioning, especially with emotional and behavioral problems. According to the 2017 NHIS survey, there was an increase of use of yoga in children ages 4–17 years old from 3.1% in 2012 to 8.4% in 2017 [17].

Various forms of yoga, including Hatha yoga, Iyengar yoga [18], Ashtanga yoga [19] as well as other mind–body interventions, [13] have been studied for gastrointestinal disorders and found to be beneficial. Yoga is thought to help by improving the biopsychosocial aspects of these conditions resulting in improvements in mental health, reduction of stress, and improved quality of life. By decreasing stress, yoga may help reduce pro-inflammatory cytokines by increasing the parasympathetic response [14] and downregulation of the hypothalamic–pituitary–adrenal axis and sympathetic nervous system. Yoga has also been shown to decrease salivary cortisol, CRP, and blood glucose in metabolic syndrome. Though the optimal frequency and duration of yoga practice is not known, studies have found that frequency trumps duration of practice as significant benefits have been demonstrated with 30–60 min of practice for 3 or more times a week for a span of 1–12 weeks [20, 21].

As a comprehensive approach to the treatment of digestive disease is necessary, we present a review of the evidence supporting the use of yoga in disorders of gut brain interaction (DGBIs) and IBD.

### Yoga in DGBIs

DGBIs are a group of disorders with chronic gastrointestinal symptoms defined by the Rome criteria without a structural, anatomic, or tissue abnormality [22, 23]. More than 50% of new pediatric GI office visits meet criteria for one of these DGBIs [24] and they carry significant physical, psychosocial, and financial burden [25]. Multiple factors are implicated in the pathophysiology of DGBIs, with a disturbance in the communication in the gut-brain axis at the center of it all. Given the variability in both the presentation and the associated factors in DGBIs, an individualized approach is needed for their treatment – one that encompasses the biopsychosocial model [26]. This encompasses a mixture of pharmacotherapy, diet, and lifestyle changes, psychological therapies, neuromodulation, and complementary and integrative approaches [27, 28]. As children with chronic conditions or greater functional disability are more likely to explore various treatment options [3], 96% of pediatric patients with functional abdominal disorders report use of at least one form of CAM [29].

Yoga has been found to be a safe and effective therapy in chronic medical conditions, [30] including DGBIs [31, 32]. The benefits of yoga seem to be particularly helpful in the pain predominant DGBIs such as functional abdominal pain (FAP), irritable bowel syndrome (IBS), and functional dyspepsia (FD). Various potential mechanisms for the role of yoga in the treatment of DGBIs have been summarized in recent reviews [32, 33]. These include a reduction in pro-inflammatory markers, reduction in anxiety and depression, enhancement of vagal tone, reduction in cortisol levels, enhanced gastro-duodenal motility, improvement in intestinal microbiota and mucosal barrier, and lessened overall pain perception.

An initial randomized trial was performed in 25 adolescent patients aged 11–18 years old who met the Rome I criteria for IBS [8]. Patients were randomly assigned to either a yoga intervention group or a control wait list control group. The yoga group received a one-hour introduction program, followed by daily practice at home with video guidance over four weeks. Both groups completed questionnaires assessing gastrointestinal symptoms, pain, functional disability, coping, anxiety, and depression at one and four weeks. The control group then received the yoga intervention and completed the questionnaires again after four weeks. Adolescents who received the yoga intervention reported lower levels of functional disability (p = 0.073), lower levels of emotion-focused avoidance (p = 0.09), lower levels of anxiety (p = 0.09), and a significant reduction in gastrointestinal symptoms (p < 0.01). Twenty-four of the 25 patients reported that they planned to continue to use yoga as part of their IBS management.

As the Rome criteria became better defined in pediatric patients and the age range for DGBIs widened, subsequent studies were added to the evidence supporting the use of yoga in the management of DGBIs. In 2011, a pilot study evaluated 20 children aged 8–18 years with FAP or IBS based on the Rome III criteria [9]. Participants were divided into two separate groups of 10 patients each, 8–11-year-olds and 11–18-year-olds, all received 10 yoga sessions over a three- month period, and completed questionnaires at several time points before, during, and after the study. Both age groups reported a statistically significant reduction in pain frequency at the end of the yoga sessions (p = 0.001) and the 8–11-year-old group also reported a significant decrease in pain intensity (p = 0.015). The decrease in pain frequency persisted at the 3-month mark, particularly for the 8–11-year-old group (p = 0.04). This study reinforced prior work and showcased some age-specific differences in the response. A third trial of 51 adolescent and young adult participants ranging from 14–26 years further demonstrated the benefits of yoga in the management of IBS [18]. Patients were randomized into either a control group or an intervention group (yoga classes twice per week over six weeks) and all patients completed the same questionnaires. Adolescents (14–17 year) in the yoga intervention group reported significantly improved physical functioning, while young adults (18–26 years) reported significant improvement in IBS symptoms, global improvement, disability, psychological distress, sleep quality, and fatigue. This again highlighted that the benefits of yoga may be age-specific and would require some individualization in clinical practice. When examined further, 50% of the adolescent group reported clinically significant improvement in abdominal pain following yoga treatment [34]. These responders also had statistically significant improvements in sleep duration and visceral sensitivity and showed trends for lower GI symptoms, functional disability, and fatigue after the yoga intervention. Qualitative interviews of the 50% of adolescents who were characterized as “non-responders” identified some factors that could impact the response to treatment, including less parental involvement and increased difficulty in keeping to the prescribed yoga treatment.

While much of the data has focused on the use of yoga in FAP and IBS, there is promising evidence in the literature to showcase its effectiveness in the treatment of other DGBIs as well. In a more recent trial, 69 patients aged 8 to 18 years with abdominal pain-related DGBIs (including FAP, IBS, FD, and abdominal migraines) were randomized into a standard medical care group without yoga intervention and a standard medical care group with yoga intervention [35]. At one year follow-up, those patients who received yoga as part of their treatment plan showed significant improvement in overall treatment success, pain intensity scores, and school attendance. Several additional cases have demonstrated reduction in symptoms scores in patients with FD [36]. There is some growing evidence for the benefits of yoga in functional constipation [20] and cyclic vomiting syndrome [37], although additional studies are required.

As with many mind–body interventions, there are some limitations associated with the use of yoga including access to and availability of trained professionals, as well as inadequate support to develop flexible individually based programs for patients. Recent studies have shown that virtual and web-based yoga sessions show similar benefits to in-person therapy [7, 38] which may help to address some of the disparities in incorporating yoga and other mind–body therapies into a treatment plan for DGBIs.

### Yoga in IBD

IBD, with primary subtypes of Crohn’s disease and Ulcerative Colitis (UC) is a chronic inflammatory autoimmune condition. Currently, IBD affects 2.39 million Americans [39] with an ever-increasing prevalence. Aside from the physically taxing aspects of IBD, patients have worse quality of life scores and higher rates of perceived stress, depression, and anxiety than patients without IBD. Baseline depression rates of 25.3% have been found in IBD and can rise to 38.9% in active disease [40]. Similarly, prevalence of anxiety in patients with quiescent IBD has been found to be as high as 32.1% and can increase to 57.6% in active disease states [40]. Furthermore, depression and anxiety are predictive of worse clinical outcomes in IBD.

In addition to increased psychosocial challenges in patients with IBD, an overlap between IBD and IBS, IBD-IBS, has been demonstrated in multiple studies. In a 2012 systematic review and meta-analysis by Halpin et al. [39], IBS prevalence in adult patients with IBD was significantly higher at 39% compared to 20% in non-IBD controls. In a 2017 cross-sectional analysis by Abdalla et al. that included 6309 adult participants with IBD, 20% of participants reported a coexisting diagnosis of IBS [41]. In a cross-sectional study in pediatric patients with IBD, prevalence of IBS-type symptoms was found to be 16.1% in those with minimal or quiescent disease, defined by a fecal calprotectin of less than 250 ug/g [42]. The overlap of these conditions supports the relevance of yoga for IBS research in the IBD population.

Considering its impact in decreasing stress, anxiety and depression, yoga has been explored as an adjunctive therapy in IBD. Two clinical trials of yoga in pediatric IBD patients have shown benefit. A 2018 pilot study by Arruda JM et al. [15], nine patients ages 10–21 years with an existing diagnosis of IBD volunteered to participate in an 8-week yoga intervention with primary outcomes of feasibility and acceptability. Yoga classes were a combination of in-person and video-based with 60 min in-person instruction/yoga classes at weeks one, three, and eight of the study period and 30-min on-line yoga classes recommended three times a week. The intervention was feasible, safe, and acceptable and all participants reported decreased stress levels, increased emotional self-awareness, and increased ability to identify and manage the physical symptoms of IBD. Limitations included small sample size, poor survey response rate, potential selection bias, and time constraints in completing at home yoga videos with only two of nine participants completing all 3-weekly on-line/at home videos. A 2023 multi-site controlled trial of yoga in pediatric IBD by Leiby et al. [12] had 78 participants from ages 10–17 years who participated in a 12-week yoga intervention. Yoga classes were offered once weekly in-person with encouragement to practice daily at home for the three-month study period. Outcomes were measured at baseline, the start and the end of the yoga intervention, and at 12 weeks after the yoga intervention. Results demonstrated significant improvements in health-related quality of life and self-efficacy particularly three months after completing the yoga class series. The overwhelming majority (93.5%) enjoyed the yoga classes with 85% of participants reporting that yoga helped with stress control and 69% that yoga helped to manage symptoms of IBD. This study was novel in that significant improvements in health-related quality of life were sustained even after the yoga intervention was completed.

Additional studies exist in adult literature, similarly demonstrating that yoga is beneficial in improving quality of life and decreasing stress and anxiety in adults with IBD. In a 2015 prospective, randomized trial by Sharma et al., 100 patients ages 16–60 with IBD in clinical remission were enrolled [14]. Patients were randomized to either an 8-week yoga intervention arm with standard IBD therapy plus a one hour in-person yoga class daily for one week followed by encouragement to practice daily for 60 min at home for the remaining seven weeks of the study period or a control arm (standard medical therapy alone). At the conclusion of the study, patients with UC randomized to the yoga intervention group reported decreased arthralgias and anxiety levels compared to the control group. In a 2017 randomized clinical trial by Cramer et al. assessing whether yoga improved disease-specific quality of life, 77 patients ages 18–70 years with UC in clinical remission but impaired quality of life were randomized into the yoga intervention group vs. the control group, who received written self-care advice [16]. The 12-week yoga intervention consisted of weekly supervised 90 min in-person yoga sessions. Outcomes of the intervention were assessed at 12 and 24 weeks and with both, patients in the yoga arm of the study demonstrated higher quality of life compared to the self-care group. Additionally, at 24 weeks, disease activity and frequency of UC flares were lower in the yoga intervention group. Finally, in a 2022 prospective, non-randomized study by Kaur et al. [11], 9 patients (mean age 52.1 years) with IBD participated in an 8-week yoga intervention study consisting of weekly in-person, 30-min yoga sessions as well as encouraged daily at home practice for the study period. Depression and mental health scores improved at week eight from baseline.

In both adult and pediatric patients with IBD, yoga has been shown to improve health-related quality of life and decrease stress and anxiety levels. Yoga is an effective adjunct to standard medical therapy in IBD. Further studies are needed regarding optimal frequency, duration of practice, and evaluation of the impact on IBD disease activity measures.

## Conclusion

IBD and DGBIs are two of the most common disorders in pediatric gastroenterology care. While IBD is primarily treated with pharmacologic therapies and DGBIs with biopsychosocial approaches, there are emerging data that show that mind–body interaction therapies such as yoga can improve patients’ symptoms and quality of life [12, 15]. This is paramount to appreciate because multiple disease states within pediatric gastroenterology require lifelong care and commitment to navigate day to day life. This burden can increase depression, anxiety, and stress. Therefore, establishing a proper routine with both pharmacologic and mind body therapies can positively influence both adherence and management of these disorders. While yoga and its specific pathophysiology impact on the central nervous system (CNS) pathways is not fully understood, several mechanisms have been proposed. Individuals who utilize yoga have been found to have increased brain-derived neurotropic factor expression, which is key in neurodevelopment and neural malleability, increased density of hippocampus, decreased amygdala volume, and increased cerebral blood flow to prefrontal cortex [43]. Some of these CNS effects, among others, are believed to directly contribute to decreased stress and anxiety.

Yoga has shown to benefit as an adjunct to standard care, but the challenge remains of how best to incorporate it into practice. Expanding options to include digital web and app-based programs in addition to in person classes with skilled yoga instructors will improve access. Also providing information to patients and families on the CNS benefits of yoga as a mind–body therapy may help increase participation. Future yoga studies in pediatric GI could investigate changes in heart rate variability, disease severity scores and biomarkers such as salivary and serum cortisol and fecal calprotectin. Although existing data has shown the benefits of yoga, continued research will help integrate this modality into routine pediatric GI care.
